# Hydrogen Sulfide Improves Angiogenesis by Regulating the Transcription of pri-miR-126 in Diabetic Endothelial Cells

**DOI:** 10.3390/cells11172651

**Published:** 2022-08-25

**Authors:** Wenlong Xue, Qingqing Zhang, Ying Chen, Yichun Zhu

**Affiliations:** 1 Department of Physiology and Pathophysiology, School of Basic Medical Sciences, Fudan University, Shanghai 200032, China; 2 Shanghai Key Laboratory of Bioactive Small Molecules, Fudan University, Shanghai 200032, China; 3 Shanghai Key Laboratory of Clinical Geriatric Medicine, Fudan University, Shanghai 200032, China

**Keywords:** hydrogen sulfide, diabetes, angiogenesis, miR-126-3p

## Abstract

Introduction: Diabetes mellitus results in high rates of cardiovascular disease, such as microcirculation disorder of the lower limbs, with angiogenesis impairment being the main factor. The endothelium functions as a barrier between blood and the vessel wall. Vascular endothelial cell dysfunction caused by hyperglycemia is the main factor leading to angiogenesis impairment. Hydrogen sulfide (H_2_S) and miR-126-3p are known for their pro-angiogenesis effects; however, little is known about how H_2_S regulates miR-126-3p to promote angiogenesis under high-glucose conditions. Objectives: The main objective of this research was to explore how H_2_S regulates the miR-126-3p levels under high-glucose conditions. Methods: We evaluated the pro-angiogenesis effects of H_2_S in the diabetic hindlimb of an ischemia mice model and in vivo Matrigel plugs. Two microRNA datasets were used to screen microRNAs regulated by both diabetes and H_2_S. The mRNA and protein levels were detected through real-time PCR and Western blot, respectively. Immunofluorescent staining was also used to assess the capillary density and to evaluate the protein levels in vascular endothelial cells. Human umbilical vein endothelial cells (HUVECs) were used in in vitro experiments. A scratch wound-healing assay was applied to detect the migration ability of endothelial cells. Methylated DNA immunoprecipitation combined with real-time PCR was chosen to identify the DNA methylation level in the HUVECs. Results: Exogenous H_2_S improved angiogenesis in diabetic mice. miR-126-3p was regulated by both diabetes and H_2_S. Exogenous H_2_S up-regulated the miR-126-3p level and recovered the migration rate of endothelial cells via down-regulating the DNMT1 protein level, which was increased by high glucose. Furthermore, DNMT1 upregulation in the HUVECs increased the methylation levels of the gene sequences upstream of miR-126-3p and then inhibited the transcription of primary-miR-126, thus decreasing the miR-126-3p level. CSE overexpression in the HUVECs rescued the miR-126-3p level, by decreasing the methylation level to improve migration. Conclusion: H_2_S increases the miR-126-3p level through down-regulating the methylation level, by decreasing the DNMT1 protein level induced by high glucose, thus improving the angiogenesis originally impaired by high glucose.

## 1. Introduction

Diabetes mellitus, a disorder of hyperglycemia caused by insulin resistance, has become a major global health pandemic [1]. In China, 110 million people suffered from diabetes in 2019, and this number is predicted to increase to 140 million by 2029 [2]. Diabetes mellitus results in high rates of cardiovascular disease. Peripheral limb ulceration and amputation arising from peripheral vascular disease are common complications of diabetes [3,4]. The endothelium functions as a barrier between the vessel wall and blood because it comprises a single cell layer lining the inner surface of the vascular lumen [5]. Endothelial cells perform multiple functions including regulation of cell adhesion, angiogenesis, inflammatory responses, vessel integrity, and vascular permeability [6]. Vascular endothelial cell dysfunction caused by hyperglycemia is the main factor leading to angiogenesis impairment [7]. Therefore, endothelial cells are the preferred target for improving angiogenesis. Although “therapeutic angiogenesis” is very challenging in the clinic, pro-angiogenic agents or gene therapy for “therapeutic angiogenesis” is still a priority scheme in patients with peripheral vascular disease.

Hydrogen sulfide (H_2_S) is the third gasotransmitter, after carbon monoxide and nitric oxide [8]. It is associated with several diseases; for instance, diabetic patients also have a lower H_2_S content in their plasma [9], and fasting blood glucose and H_2_S levels are negatively correlated in streptozotocin-induced diabetic rats [10]. Since Cai et al. first reported that H_2_S promotes angiogenesis in 2007 [11], H_2_S has become of increasing interest. Over the past decade, H_2_S has been discovered to improve skin wound healing in diabetic mice via anti-inflammation or antioxidants [12,13], particularly in modulating angiogenesis [14,15]. However, the pro-angiogenic mechanisms of H_2_S in diabetes remain to be further researched.

MicroRNAs are a class of non-coding RNAs, 18–22 nucleotides in length. They also regulate diabetic wound healing through pro-angiogenesis, e.g., miR-615-5p and miR-92a [16,17]. miR-126-3p is well-known for regulating angiogenesis in vascular endothelial cells [18,19,20,21,22]. miR-126 deficiency in mice leads to the formation of fragile and leaky vessels, aberrant endothelial tube hierarchy, and impaired endothelial cell migration and proliferation [18,23]. miR-126-3p can suppress its target gene PI3KR2, as well as SPRED1 and the like, to increase angiogenesis [24]. Interestingly, H_2_S also regulates miRNA transcription, and the crosstalk between H_2_S and microRNAs plays a crucial role in the pathophysiology of cardiovascular disease [25]. There are several works reporting that H_2_S can decrease the cardiomyocyte apoptosis induced by ischemia/reperfusion or can fight Parkinson’s disease by regulating microRNAs [26,27]. However, the relationship between H_2_S and microRNAs in regulating angiogenesis under high-glucose conditions remains unclear.

DNA methylation is an important epigenetic modification to regulate gene expression. DNA methyltransferase 1 (DNMT1) is one of the proteins maintaining DNA methylation. In colorectal cancer cells, DNMT1 regulates thymosin β 10 (TMSB10) expression to decrease tumor growth via maintaining the methylation of miR-152-3p [28]. In addition, DNMT1 acts as an anti-angiogenic protein [29,30]. The DNMT1 protein level is also connected with diabetes; for example, Zhang et al. found that DNMT1 protein is highly expressed in podocytes upon high-glucose treatment or in diabetic mice. Additionally, DNMT1 is a potential target for attenuating podocyte injury and diabetic nephropathy [31].

In our previous study, we found that H_2_S can rescue the miR-126-3p level by down-regulating the DNMT1 protein level in human umbilical vein endothelial cells (HUVECs) [32]. However, we remain unaware of how DNMT1 decreases miR-126-3p upon high-glucose treatment, and whether DNMT1 has an effect on blood flow recovery in diabetic hindlimb ischemia mice regulated by H_2_S. Herein, we aimed to explore the mechanism responsible for how H_2_S regulates the miR-126-3p level to improve the function of endothelial cells and to promote blood flow in diabetic hindlimb ischemia mice. In addition, HUVECs were also used in in vitro experiments.

## 2. Materials and Methods

### 2.1. Modeling of Type I Diabetic Mice

Wild-type C57BL/6 male mice (22–24 g) were obtained from the SLAC Laboratory (Shanghai, China), and the mice were maintained on a 12 h light/dark cycle with food and water available ad libitum. After acclimatization for two weeks, the mice were rendered diabetic by an intraperitoneal dose of 50 mg/kg of streptozotocin (Sigma-Aldrich, Saint Louis, MO, USA) for five consecutive days. After two weeks, blood glucose fasting for 4 h was detected via the tail of the animal using a portable glucometer (Johnson, New York, NY, USA). Mice with blood glucose levels of >13.8 mmol/L were categorized into the type I diabetic mice group. The mice without STZ treatment were considered as the non-diabetic control group. In this study, we chose only male mice because estrogen directly modulates angiogenesis via effects on endothelial cells [33], which may disrupt the pro-angiogenesis effect of H_2_S.

### 2.2. Murine Hindlimb Ischemia Model

The murine hindlimb ischemia model was set up as described previously [34]. Briefly, the C57BL/6 male mice were anesthetized with 1% pentobarbital sodium and placed on a heating pad (37 °C) to maintain body temperature. Then, the femoral artery was ligated and arteriotomy was performed without damaging the vein or nerve along the inner left hindlimb. Femoral artery ligation was not performed on the mice treated with a sham operation. The blood flow of the mice was monitored immediately using a MoorLDI2-2 laser Doppler imaging system (Moor Instruments, Devon, UK), to authenticate whether the model was successful. Only the mice that had been operated on successfully (the ratio of measurements in the ligated and non-ligated contralateral limbs was lower than 0.1) were used in the following experiment. NaHS (30 and 60 μmol/kg/day; Sigma, Saint Louis, MO, USA) was intraperitoneally injected every day for 14 days after ischemia [35]. Equivoluminal injections of saline solution served as the vehicle.

### 2.3. Immunofluorescence Assay

The gastrocnemius muscles were fixed in 4% paraformaldehyde and embedded in paraffin. For immunofluorescence staining, the embedded samples were sectioned, and these sections (4 μm) were deparaffinized and rehydrated using dimethylbenzene, graded ethanol, and water baths. Antigens were retrieved using sodium citrate buffer for 10 min at 95 °C. The sections were washed three times with PBS. Afterward, the sections were permeabilized with 0.01% Triton X-100 for 15 min. After washing three times with PBS, the sections were incubated in PBS containing 5% goat serum for 2 h to avoid non-specific protein binding. The sections were incubated with primary CD31 (mouse monoclonal antibody, 1:100; Novus, Littleton, CO, USA) and/or DNMT1 (rabbit polyclonal antibody, 1:100; Affinity, USA) overnight at 4 °C and further stained with Alexa Fluor^®^ 488 anti-rabbit IgG (H+L) (1:100; CST, USA) and/or Alexa Fluor^®^ 594 anti-mouse IgG (H+L) (1:100; CST, Topsfield, MA, USA) for 2 h at room temperature. After washing three times with PBS, 6-diamidino-2-phenylindole (DAPI; Beyotime, Shanghai, China) was used to visualize nuclear localization. Finally, the sections were immediately examined using a confocal microscope (ZEISS, Jena, Germany). The data were analyzed using ZEN 2010.

### 2.4. GEO Datasets of MicroRNA between the Diabetic and Control Groups

A high throughput dataset (accession number GSE140959) comparing the plasma microRNA expression profiles between control human (10 samples) and type II diabetic patients (10 samples) was downloaded from the GEO database [36]. These microarray data of GSE140959 were based on the platform GPL16384 (Affymetrix Multispecies miRNA-3 Array).

### 2.5. Data Preprocess and Differential MicroRNA Expression Analysis

To identify whether the microRNAs were significantly dysregulated in type II diabetes mellitus compared to the controls, differential microRNA expression analyses were conducted using the web analysis tool GEO2R. A volcano plot of the whole dataset was analyzed using the R-package ggplot2 (RStudio) and was generated using GraphPad Prism 7. An absolute log_2_ (fold change) of >1.5 and a −log (*p*-Value) of >1.3 were considered as the criteria of significantly differently expressed genes.

### 2.6. Affymetrix MicroRNA Profiling

To detect the microRNA expression in primary human umbilical vein endothelial cells (HUVECs), the HUVECs were treated with NaHS (30 μmol/L) or the vehicle for 12 h in the first instance. Then, the RNA was isolated from the HUVECs using Trizol (Thermo, Waltham, MA, USA), in accordance with the protocol of the manufacturer. The microRNA expression was tested by an Affymetrix Gene Chip Expression Assay (Affymetrix, Santa Clara, CA, USA).

### 2.7. Reverse Transcription and Real-Time PCR

The RNA isolated from the tissues or HUVECs was measured by Nanodrop (Thermo, Waltham, MA, USA) to detect the concentration. Then, 1 μg of RNA was reverse transcribed using a FastKing Reverse Transcription Kit with gDNase (TIANGEN, Beijing, China). Gene amplification was achieved by real-time PCR using an SYBR Green Real-Time PCR Kit (Toyobo, Osaka, Japan). The reverse transcription of miR-126-3p was achieved by stem-loop RT-PCR. The sequences for the reverse and real-time PCR primers are summarized in Table S1. The 7300 Real-Time PCR System (Applied Biosystems, Waltham, MA, USA) was used for cDNA amplification and detection.

### 2.8. In Vivo Matrigel Plugs Analysis

Before Matrigel plugs analysis, the mice were divided into four groups: the non-diabetic control and vehicle group, diabetes and vehicle group, diabetes and NaHS (30 μmol/kg/day) group, and diabetes and NaHS (60 μmol/kg/day) group. On the first day, each mouse was subcutaneously injected with 500 μL of Matrigel plugs and was intraperitoneally injected saline solution or NaHS (30 and 60 μmol/kg/day) for seven consecutive days. The mice were euthanized after day 7, and the Matrigel plugs were harvested to detect the hemoglobin content and RNA levels, as described previously [37,38]. Briefly, the Matrigel plugs were recovered by dissection. The hemoglobin content was measured using the tetramethylbenzidine (TMB) method, and the values were normalized by the weight of the plugs.

### 2.9. Cell Culture

The HUVECs were purchased from ALLCELLS (Shanghai, China). The HUVECs from passages 4 to 7 were used in the experiment and were cultured in endothelial completed medium (ALLCELLS, Shanghai, China) at 37 °C in 5% CO_2_. The HUVECs were cultured in normal glucose medium (NG; 5.5 mmol/L of D-glucose) or high-glucose medium (HG; 33.3 mmol/L of D-glucose) for 48 h, and the L-glucose (27.8 mmol/L) contained in NG was used as the osmotic pressure control.

### 2.10. Cell Transfection and Lentivirus Infection

The antagomir NC and antagomir miR-126-3p were purchased from RIBOBIO Inc. (Guangzhou, China). The HUVECs were transfected in 150 μmol/L of antagomir miR-126-3p to inhibit the endogenous miR-126-3p level with Lipofectamine RNAiMAX Transfection Reagent (Thermo, Waltham, MA, USA), in accordance with the protocol of the manufacturer, and antagomir NC was used as the control. DNMT1-shRNA or CSE-GFP lentivirus was packaged by our laboratory according to a previous study [32]. After the lentivirus was prepared, the HUVECs were infected with lentivirus for 8 h, and 10 μg/mL of polybrene (Sigma, Saint Louis, MO, USA) was also added to increase the infection efficiency. Fresh complete medium was added to replace the lentivirus and polybrene-containing medium 8 h later. NC-shRNA and GFP-expressing lentivirus were used as the control for DNMT1-shRNA and CSE-GFP lentivirus, respectively. In addition, another CSE overexpression lentivirus (OE-CSE), without a GFP label, was used to avoid interference with the HSip-1 DA probes when detecting the cellular H_2_S level.

### 2.11. Measurements of the H_2_S Level

The endogenous H_2_S level in the HUVECs was measured using HSip-1 DA probes (DOJINDO, Kumamoto, Japan), in accordance with the protocol of the manufacturer [39]. Briefly, the HUVECs were washed with HBSS three times before being treated with the HSip-1 DA probes; then, the HUVECs were incubated with HSip-1 DA probes for 0.5 h. After 0.5 h, the HUVECs were washed three times with HBSS again to remove any unbound probe. Then, 1 mL of HBSS was added to the confocal dish as an imaging buffer. The cellular H_2_S level was immediately detected by a confocal microscope (ZEISS, Jena, Germany). The fluorescence intensity is represented by the cellular H_2_S level and was analyzed by ImageJ software.

### 2.12. Scratch Wound-Healing Assay

The HUVECs were grown to confluence (90%) in pre-coated six-well plates and starved for 12 h before the experiments for synchronous growth. Sterile pipette tips (1000 μL) were used to make scratch wounds, and the cells were washed three times with PBS to remove any floating cells. Then, the remaining cell sheets were cultured in basal medium (1% FBS) for another 24 h with different treatments. Images of the wounded area were taken immediately after the scratch and 24 h later. The wound areas were measured using ImageJ software.

### 2.13. Methylated DNA Immunoprecipitation (MeIP)

MeIP was performed as detailed previously [40]. Briefly, genomic DNA was isolated from the HUVECs in the first instance, and then genomic DNA was sonicated to obtain 300–100 bp fragments. The denatured DNA fragments (800 ng) were incubated with 5-mC antibody (1:50; Abcam, Waltham, MA, USA) for 2 h at 4 °C and washed three times with IP buffer (10 mM NaPO_4_, 140 mM NaCl, 0.05% Triton X-100, pH 7.0), then Dynabeads (30 μL; Thermo, Waltham, MA, USA) were added into the mix with the DNA fragments and 5-mC antibody. The sample was then incubated in a rotating tube holder overnight at 4 °C. The DNA fragments bound with the 5-mC antibody were purified with Proteinase K and phenol/chloroform. The purified DNA fragments were then detected using real-time PCR to evaluate the DNA methylation level. The primer sequences for the real-PCR are listed in Table S1.

### 2.14. Ethics Approval and Consent to Participate

Our animal experimental procedures were performed according to the Guide for the Care and Use of Laboratory Animals of the National Institutes of Health (NIH) of the United States and were approved by the Ethics Committee for Experimental Research of Fudan University, School of Basic Medical Sciences. IACUC Animal Project Numbers (APN): 20180302-026 and 20180302-045.

### 2.15. Statistical Analysis

The data were presented as mean ± SEM. All experiment data were analyzed using SPSS software and performed with GraphPad Prism 7 software. Two treatment groups were compared with student’s t-test, and three or more treatment groups were tested by one-way ANOVA followed by LSD tests. Probability value <0.05 was considered statistically significant.

## 3. Results

### 3.1. Exogenous H_2_S Improved Angiogenesis in Diabetic Mice

**Figure 1:**
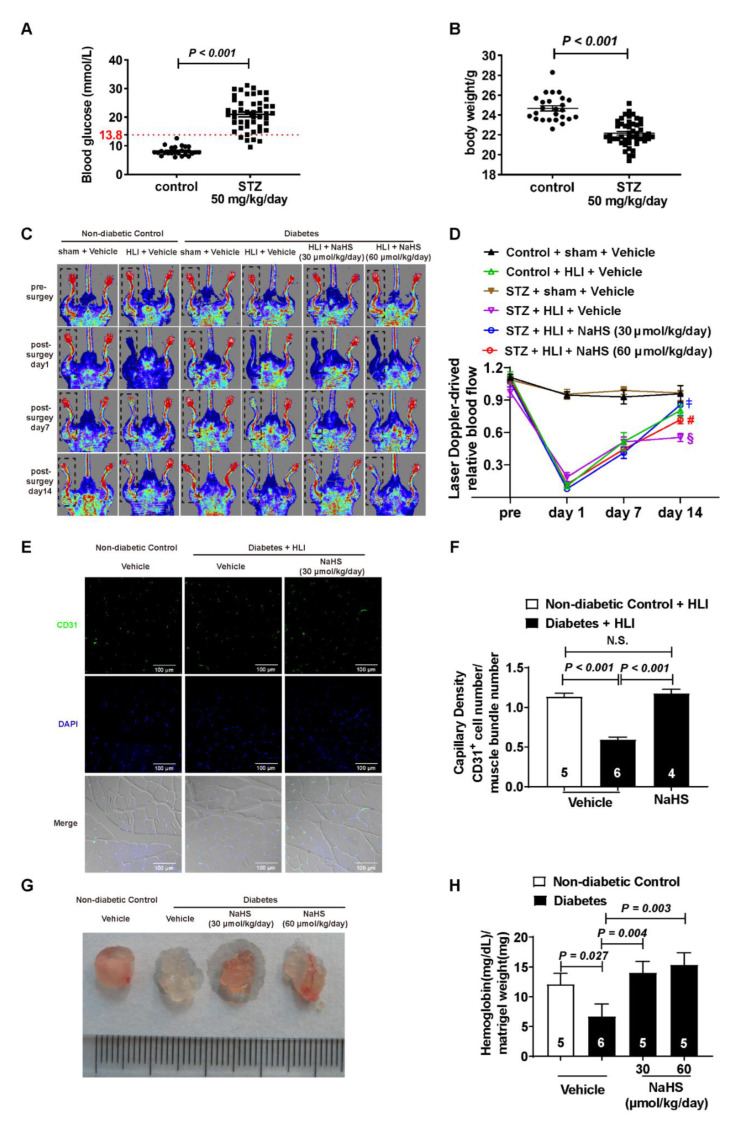
Exogenous H_2_S promoted angiogenesis in vivo. (**A**) Blood glucose fasting in mice for 4 h before and after STZ (50 mg/kg/day) treatment every day for five days. (**B**) Body weight of the mice before and after STZ (50 mg/kg/day) treatment every day for five days. (**C**,**D**) Representative blood flow images and statistical analysis with or without NaHS treatment in diabetic hindlimb ischemia mice and non-diabetic mice; n = 5~9. § vs. control and HLI and vehicle; ‡, # vs. STZ and HLI and vehicle. (**E**,**F**) Representative micrographs showing the capillary density stained with anti-mouse CD31 antibodies and statistical analysis with or without NaHS treatment in diabetic hindlimb ischemia mice and non-diabetic mice; bar = 100 μm, n = 4~6. (**G**,**H**) Representative Matrigel plug images and hemoglobin content in Matrigel plugs with or without NaHS treatment in diabetic and non-diabetic mice; n = 5~6.

To determine whether type I diabetic mice were created successfully, blood glucose fasting for 4 h was detected after the mice were treated with 50 mg/kg of STZ for five days. We chose diabetic mice with a blood glucose fasting that for 4 h was higher than 13.8 mmol/L for the next experiment (Figure 1A and Table S2), and these mice also showed lower body weight compared to the non-diabetic controls (Figure 1B and Table S3). The diabetic mice treated with NaHS (30 and 60 μmol/kg/day) for 14 days showed greater blood-flow recovery compared to the diabetic mice treated with the vehicle in hindlimb ischemia model (HLI) (Figure 1C,D). Hindlimb ischemia did not affect the gastrocnemius muscle weight in either the diabetic or non-diabetic mice (Figure S1A). However, the weight of the non-ischemic gastrocnemius muscle decreased in the diabetic mice compared to the non-diabetic mice (Figure S1B). We also observed the capillary density through immunofluorescence, and our results indicated that the diabetic mice had lower capillary density in the ischemic gastrocnemius muscles compared to the non-diabetic control mice; however, exogenous H_2_S improved the capillary density inhibition induced by diabetes (Figure 1E,F). Additionally, an in vivo Matrigel plug assay was also used to evaluate the pro-angiogenesis effect of H_2_S. Our results showed that the hemoglobin content was inhibited in the Matrigel plugs of the diabetic mice compared to the non-diabetic mice and that NaHS treatment recovered the hemoglobin content in the diabetic mice (Figure 1G,H).

### 3.2. MicroRNAs were Regulated by Both Diabetes and H_2_S

**Figure 2:**
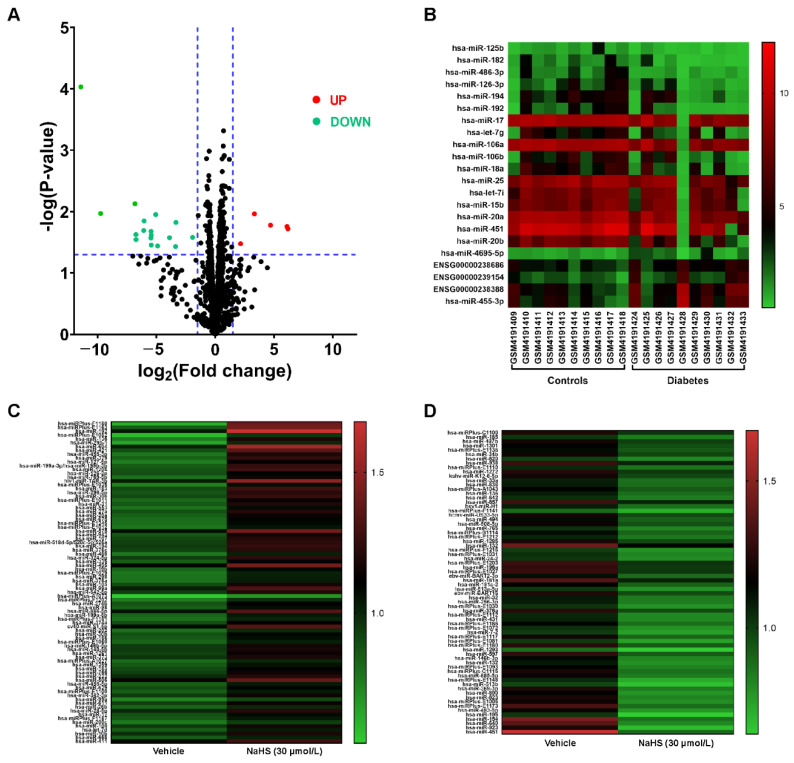

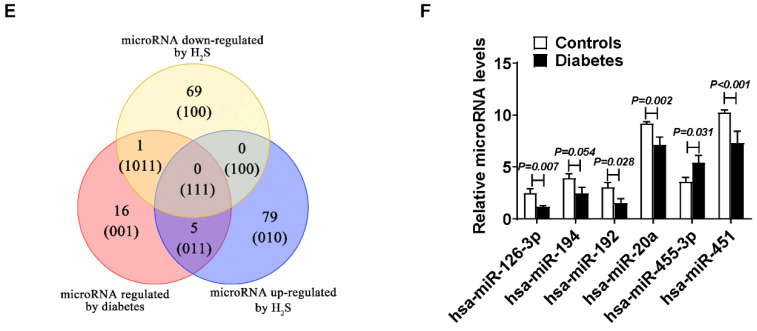
The microRNAs regulated by diabetes and exogenous H_2_S. (**A**) Volcano plot of the expressed microRNAs in the plasma between the control and diabetic patients; n = 10. (**B**) Heat map of the expression levels of 22 dysregulated microRNAs. Each row represents a microRNA and each column represents a sample. (**C**) Heat map of the microRNA expression levels up-regulated by NaHS (30 μmol/L). (**D**) Heat map of the microRNA expression levels down-regulated by NaHS (30 μmol/L). (**E**) Venn diagram of the significantly upregulated or downregulated microRNAs between diabetes-induced dysregulated microRNAs and H_2_S-regulated microRNAs. (**F**) Statistical analysis of the dysregulated microRNA expression in plasma induced by diabetes and also regulated by H_2_S; n = 10.

In order to find out whether microRNAs are related to diabetes, we analyzed the plasma microRNA expression profiles between type II diabetic patients and healthy humans to identify whether microRNAs are significantly dysregulated in type II diabetes mellitus (Excel S1). We found that 22 microRNAs were dysregulated: 5 microRNAs were up-regulated and 17 were down-regulated (Figure 2A,B). Combined with the microRNAs regulated by H_2_S in the HUVECs (Figure 2C,D), we found that six microRNAs were regulated by both diabetes and H_2_S, and one of them was up-regulated, while the others were downregulated (Figure 2E,F). miR-126-3p is one of the microRNAs that decreased in the plasma of the diabetic patients but was up-regulated by H_2_S.

### 3.3. Exogenous H_2_S Regulated the miR-126-3p and DNMT1 Levels in the Ischemic Gastrocnemius Muscles of Diabetic Mice

**Figure 3:**
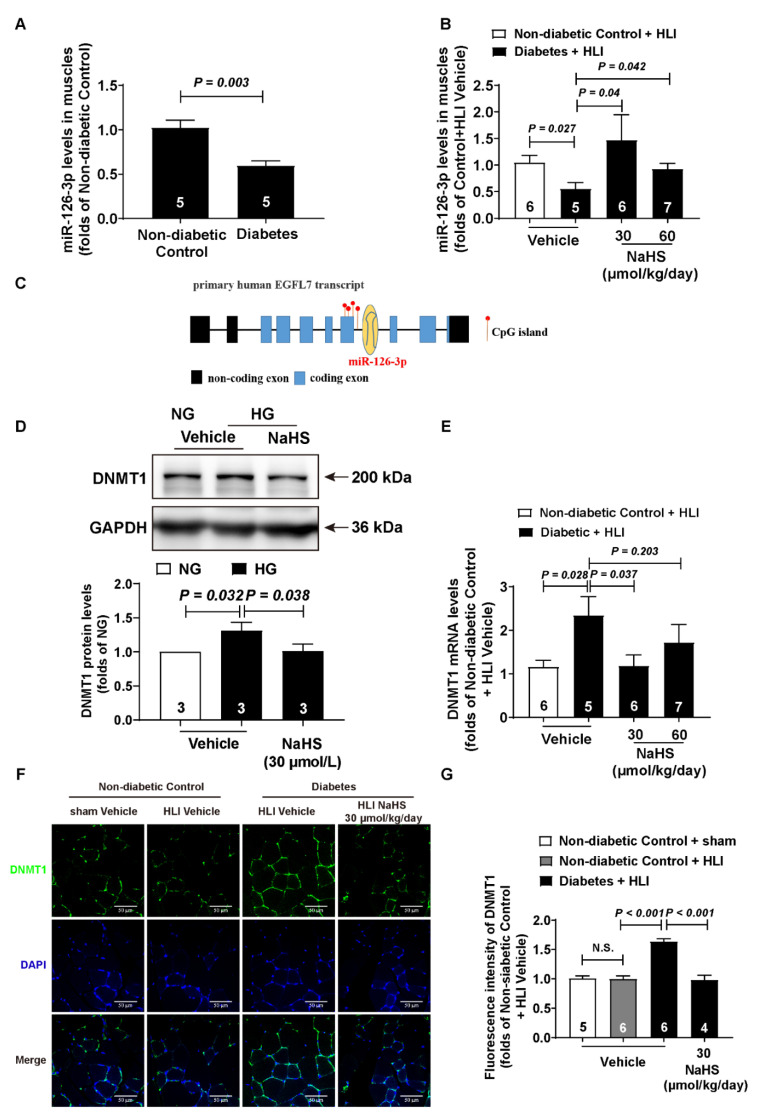
Exogenous H_2_S enhanced the miR-126-3p level and decreased the DNMT1 level in the gastrocnemius muscles of diabetic mice. (**A**) miR-126-3p levels in diabetic muscles compared to the non-diabetic controls; n = 5. (**B**) miR-126-3p levels in the ischemia muscles of diabetic mice with NaHS (30 and 60 μmol/kg/day) treatment compared to the non-diabetic controls; n = 5~7. (**C**) Schematic illustration of CpG islands before the gene of miR-126-3p. (**D**) DNMT1 protein level of HUVECs with NaHS (30 μmol/L) treatment under high-glucose conditions; n = 3. (**E**) DNMT1 mRNA level in the ischemia muscles of diabetic mice with NaHS (30 and 60 μmol/kg/day) treatment compared to the non-diabetic controls; n = 5~7. (**F**,**G**) Representative micrographs showing DNMT1 with rabbit antibodies against DNMT1 and statistical analysis of the DNMT1 protein expression in the gastrocnemius muscles of diabetic hindlimb ischemia mice compared to non-diabetic mice with or without NaHS (30 μmol/kg/day) treatment; bar = 50 μm, n = 4~6.

To confirm whether the miR-126-3p level decreased in the diabetic mice, we detected the miR-126-3p level in the gastrocnemius muscles. Our results indicated that the miR-126-3p level in the gastrocnemius muscles decreased in the diabetic mice compared to the non-diabetic mice (Figure 3A). Furthermore, NaHS treatment partly rescued the miR-126-3p level in the ischemic gastrocnemius muscles (Figure 3B). So, to explore how H_2_S regulates the miR-126-3p level, we analyzed the base sequence around miR-126-3p through MethPrimer software and found that there are several CpG islands upstream of miR-126-3p (Figure 3C), many of which affected the transcription of primary miR-126 (pri-miR-126). As DNMT1 is one of the significant proteins regulating DNA methylation, we detected the DNMT1 protein level in the HUVECs and ischemic gastrocnemius muscles. Exogenous H_2_S rescued the DNMT1 protein level in the HUVECs induced by high glucose (Figure 3D). Furthermore, both the DNMT1 mRNA (Figure 3E) and protein levels (Figure 3F,G) increased in the ischemic gastrocnemius muscles, and NaHS treatment rescued this phenomenon. Meanwhile, ischemia treatment had no effect on the expression of DNMT1 (Figure 3F,G).

### 3.4. miR-126-3p Was Downstream of DNMT1 to Regulate Angiogenesis in Endothelial Cells

**Figure 4:**
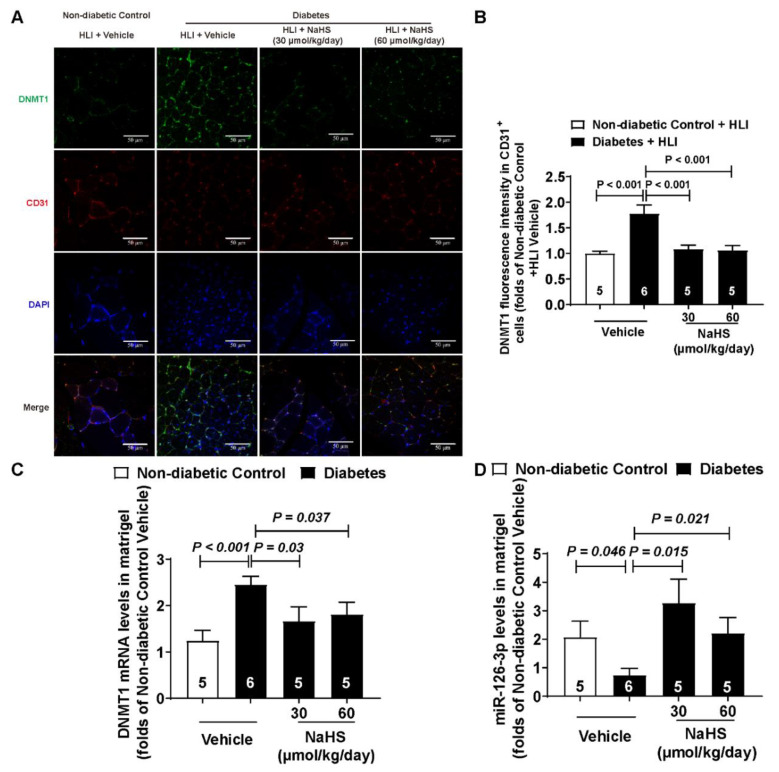

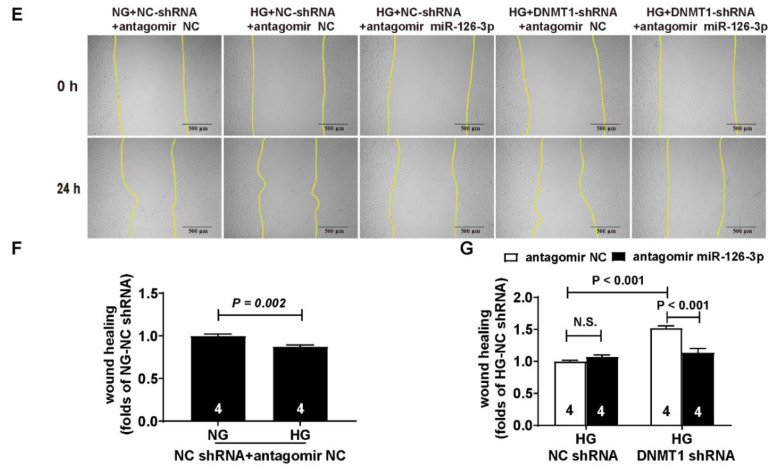
Exogenous H_2_S enhanced the miR-126-3p level and decreased the DNMT1 level in endothelial cells. (**A**) Representative micrographs showing the endothelial cells in the gastrocnemius muscles, 14 days after hindlimb ischemia surgery, stained with mouse antibodies against CD31 and rabbit antibodies against DNMT1; bar = 50 μm. (**B**) Statistical analysis of the DNMT1 protein expression in the endothelial cells of the gastrocnemius muscles with or without NaHS (30 and 60 μmol/kg/day) treatment; n = 5~6. (**C**) DNMT1 mRNA level in Matrigel plugs with or without NaHS (30 and 60 μmol/kg/day) treatment; n = 5~6. (**D**) miR-126-3p level in Matrigel plugs with or without NaHS (30 and 60 μmol/kg/day) treatment; n = 5~6. (**E**) Representative micrographs of HUVEC scratch wound healing with DNMT1 and miR-126-3p knocked down under high-glucose conditions; bar = 500 μm. (**F**) Statistical analysis of HUVEC scratch wound healing treated with high glucose; n = 4. (**G**) Statistical analysis of HUVEC scratch wound healing with DNMT1 and miR-126-3p knocked down under high-glucose conditions; n = 4.

Considering that the progress of angiogenesis mainly occurs in endothelial cells, we detected the DNMT1 protein level in the endothelial cells of the ischemic gastrocnemius muscles through immunofluorescence. The results indicated that exogenous H_2_S decreased the DNMT1 protein level in the endothelial cells that were up-regulated (induced by diabetes) (Figure 4A,B). The Matrigel plug model is the most classic model used to evaluate the effect of angiogenesis, and the main cells that grow into Matrigel plugs are endothelial cells. Thus, we detected the DNMT1 and miR-126-3p levels in the Matrigel plugs. The DNMT1 mRNA level in the Matrigel plugs was in accordance with the protein levels in the endothelial cells of the ischemic gastrocnemius muscles (Figure 4C). Moreover, exogenous H_2_S also increases the miR-126-3p level in the Matrigel plugs, which was decreased by diabetes (Figure 4D). Moreover, to confirm whether DNMT1 and miR-126-3p take part in angiogenesis under high-glucose conditions, we knocked down DNMT1 and miR-126-3p. The inhibition efficiency of the DNMT1 protein and miR-126-3p levels were approximately 84% and 30%, respectively (Figure S2A–C). Our results demonstrated that high-glucose-inhibited wound healing (Figure 4E,F), and DNMT1 knocked down under high-glucose conditions improved the wound healing of endothelial cells (Figure 4E,G). However, the effect of pro-migration was blocked, while DNMT1 and miR-126-3p were knocked down at the same time (Figure 4E,G).

### 3.5. CSE Overexpression Increased the Transcription of pri-miR-126 under High-Glucose Conditions

**Figure 5:**
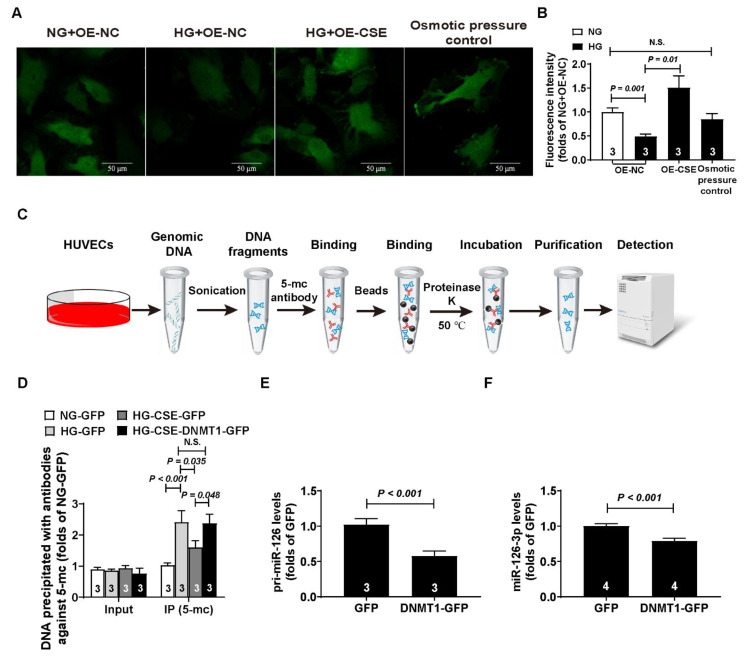
DNMT1 overexpression decreased pri-miR-126 and miR-126-3p levels in endothelial cells. (**A**) Representative micrographs showing the endogenous H_2_S in HUVECs with or without CSE overexpression under high-glucose conditions; bar = 50 μm. (**B**) Statistical analysis of fluorescence intensity of endogenous H_2_S; n = 3. (**C**) Schematic illustration of methylated DNA immunoprecipitation. (**D**) Statistical analysis of methylated DNA immunoprecipitation; n = 3. (**E**) pri-miR-126 levels in HUVECs with DNMT1 overexpression; n = 3. (**F**) miR-126-3p levels in HUVECs with DNMT1 overexpression; n = 4.

CSE is the main synthetase in the cardiovascular system, so we enhanced the endogenous H_2_S level through overexpression of the CSE protein. We overexpressed the DNMT1 protein level to simulate the effects induced by high glucose and rescued via CSE overexpression. The DNMT1 and CSE overexpression efficiencies were evaluated by Western blot, and they were severally up-regulated by 1.4- and 6-fold, respectively (Figure S2D–F). Our results showed that CSE overexpression increases the cellular H_2_S level in the HUVECs, which was decreased by high-glucose treatment (Figure 5A,B). MeIP combined with real-time PCR was used to detect the DNA methylation levels, according to the protocol (Figure 5C). The DNA methylation level was increased under high-glucose conditions (Figure 5D). CSE overexpression decreased the DNA methylation level induced by high glucose. However, the effects of CSE were blocked, while DNMT1 was overexpressed at the same time (Figure 5D). Considering that the DNMT1 protein level was up-regulated by high glucose, we overexpressed the DNMT1 protein level to simulate this effect. We found that the pri-miR-126 and miR-126-3p levels declined, while DNMT1 was overexpressed (Figure 5E,F).

### 3.6. CSE Overexpression Rescued the miR-126-3p Level through Regulating DNMT1 Expression

**Figure 6:**
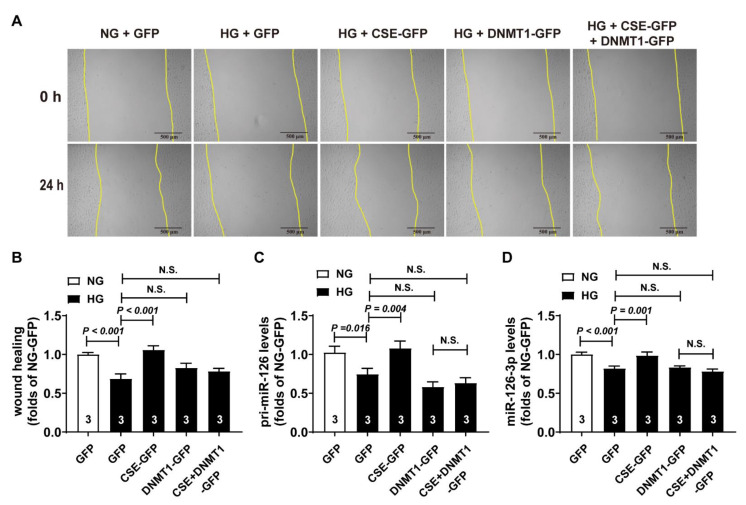
Upregulation of endogenous H_2_S increased the transcription of pri-miR-126 via decreasing the DNMT1 level in endothelial cells. (**A**,**B**) Representative images of scratch wound healing in HUVECs with CSE or DNMT1 overexpression (**A**) and statistical analysis (**B**); bar = 500 μm, n = 3. (**C**) pri-miR-126 level in HUVECs with CSE or DNMT1 overexpression; n = 3. (**D**) miR-126-3p level in HUVECs with CSE or DNMT1 overexpression; n = 3.

To determine whether endogenous H_2_S improves the cell migration impaired by high glucose, we simultaneously overexpressed the CSE and DNMT1 protein levels. Our results exhibited that CSE partially rescued the migration inhibited by high glucose, and this effect was blocked while DNMT1 was overexpressed (Figure 6A,B). Likewise, high-glucose-induced downregulation in pri-miR-126 and miR-126-3p was rescued with CSE overexpression, and these effects were blunted in HUVECs while DNMT1was overexpressed (Figure 6C,D).

## 4. Discussion

Diabetes mellitus is one of the primary threats to human health. It induces microvascular complications caused by vascular endothelial cell dysfunction. Gangrene and diabetic retinopathy are two of the most representative diseases resulting from diabetes mellitus, and both of these diseases are induced by abnormal angiogenesis. The low ability of angiogenesis ulteriorly causes microcirculation disorders in the lower limbs. As a pro-angiogenesis gasotransmitter, the H_2_S level is lower in the plasma of individuals with diabetes mellitus [41], and it is likely to improve angiogenesis through regulating vascular endothelial cells in hyperglycemia.

The hindlimb ischemia model is a classic in vivo model used to evaluate the function of angiogenesis [42]. Our results displayed that H_2_S improved the blood flow in hindlimb ischemic diabetic mice. Considering that capillaries play an important role in angiogenesis, we detected and found that H_2_S increased the capillary density. We also assessed the pro-angiogenesis effects of H_2_S through a Matrigel plug assay. Our results suggest that H_2_S rescues the hemoglobin in Matrigel plugs down-regulated by hyperglycemia, which is consistent with the previous study [43]. Although H_2_S improved the blood supply via promoting angiogenesis in diabetic mice, little is known about the mechanism by which this occurs.

MicroRNAs are a class of endogenous small non-coding RNAs, which can induce mRNA degradation and translational repression via interaction with the 3′ UTR, 5′ UTR, or CDS of the target mRNAs [44]. Given that abnormally expressed microRNAs play a critical role in the pathogenesis of microvascular complications [45], we focused on whether microRNAs participate in the angiogenesis regulated by H_2_S. We found that miR-126-3p is regulated by both diabetes and H_2_S, by analyzing two sets of microRNA array data. Zhang et al. reported that miR-126-3p could be a biomarker of diabetes mellitus, showing significantly reduced expression in diabetic plasma [46], which further indicates that the microRNA array data from GEO databases are reliable. In diabetic mice, we also confirmed that the miR-126-3p level was down-regulated when induced by STZ, and exogenous H_2_S improved the miR-126-3p level in the muscle. Considering the data of plasma microRNA from patients with type II diabetes, we thought the high-fat model of type II diabetes is indeed more relevant. However, it would be better if we measured the microRNA levels in mouse plasma.

DNA methylation, an important epigenetic modification, regulates a number of biological processes. Ohad Glaich reported that DNA methylation can regulate the generation of microRNAs [47]. Jyotirmaya Behera et al. found that H_2_S decreases DNMT activity and then alleviates HHcy-induced hypermethylation [48]. We reported the phenomenon that H_2_S can rescue the miR-126-3p level via inhibiting DNMT1 expression in the HUVECs [32], and exogenous H_2_S reduces the DNMT1 mRNA and protein levels in muscle-induced hyperexpression. Vascular endothelial cells are the main cells participating in angiogenesis [49]. We detected the DNMT1 protein level in CD31^+^ cells via immunofluorescent staining, and we found that H_2_S decreased the DNMT1 levels in vascular endothelial cells up-regulated by hyperglycemia. However, it is difficult for us to isolate vascular endothelial cells to detect the DNMT1 mRNA and miR-126-3p levels in ischemic muscles; thus, we examined the endothelial cells in Matrigel plugs, where endothelial cells are newly formed [32]. Exogenous H_2_S also recovered the DNMT1 mRNA and miR-126-3p levels in endothelial cells under high-glucose conditions. It is worth noting that several figures, such as Figure 3B,E, showed that increased H_2_S reverses any beneficial effects. In fact, there is no significant difference between 30 and 60 μmol/kg/day of NaHS in diabetic mice because of the large dispersion in the data. H_2_S ameliorates angiogenesis in diabetic mice with no dose-dependence, but it is unknown whether a higher concentration of H_2_S would show beneficial effects. Moreover, the number of CD31 positive cells and the content of hemoglobin in the Matrigel plugs are usually used to evaluate angiogenesis. In this study, we only detect the hemoglobin content in the Matrigel plugs. We believe that it will be more convincing if we measure the CD31 positive cells via HE staining or immunohistochemical staining with CD31 antibody at the same time. Furthermore, we also confirmed that miR-126-3p mediates the inhibitory effect of DNMT1 on endothelial cell migration. Nevertheless, we still do not know how DNMT1 mediates the process of H_2_S regulating the miR-126-3p level under high-glucose conditions. In addition, we do not know how H_2_S decreases the DNMT1 mRNA level. mRNA production is regulated by transcription factors and transcription repressors, and H_2_S can regulate the expression of transcription factors [50,51]. Considering H_2_S decreases the DNMT1 protein level via limiting its mRNA, we think H_2_S regulates DNMT1 at the transcriptional level.

CpG islands or residues, the significant sites in which methylation occurs, are located in the host gene, intronic miRNA promoter, and miRNA gene promoters. DNA methylation affects microRNA expression via reducing these promoters’ activities [52]. We further analyzed the features of the miR-126-3p sequence using MethPrimer software, and found that miR-126-3p and pri-miR-126 are located in primary EGFL7 transcription. Additionally, several CpG islands were found upstream of pri-miR-126, indicating that miR-126-3p expression may be affected by DNA methylation. To confirm the degree of DNA methylation upstream of pri-miR-126, we used DNA MeIP combined with real-time PCR. Here, we overexpressed DNMT1 expression to simulate the effects of high glucose on endothelial cells. The results indicated that high glucose and DNMT1 overexpression increased the DNA methylation level in the HUVECs, in accordance with the previous study [53]. Moreover, the levels of pri-miR-126 and miR-126 were inhibited with DNMT1 overexpression. We also found that CSE overexpression inhibited the DNA methylation level induced by high glucose and rescued pri-miR-126 transcription and miR-126-3p production. However, the effects of CSE were blocked while DNMT1 was overexpressed. In addition, DNMT1 mediated the endothelial cell migration regulated by H_2_S.

The novelty is that we first demonstrated that H_2_S improves angiogenesis, by regulating the transcription of the pri-miR-126 and miR-126-3p levels in diabetic endothelial cells, via down-regulating high-glucose-mediated DNMT1 upregulation.

## 5. Conclusions

In conclusion, this study focused on the role of H_2_S in the improvement of angiogenesis in diabetic mice via down-regulating the DNA methylation level. DNMT1 plays a critical role during the angiogenesis process in diabetic mice. Our findings provide new insights into the role of H_2_S in regulating miR-126-3p under high-glucose conditions and suggest a new target for ischemic therapy in diabetes mellitus.

## Data Availability

All supporting data and materials are available online.

## Supplementary Materials

The following supporting information can be downloaded at: https://www.mdpi.com/article/10.3390/cells11172651/s1, Figure S1: Muscle weight of the diabetic mice; Figure S2: The efficiency of manipulating the DNMT1, miR-126-3p, and CSE expression in endothelial cells; Table S1: Sequences for the reverse and real-time PCR primers; Table S2: Blood glucose with or without STZ treatment; Table S3: Body weight with or without STZ treatment; Excel S1: miRNAs expression in plasma between healthy controls and diabetes mellitus.
