# Supplementary material for: Hydrogen Sulfide Improves Angiogenesis by Regulating the Transcription of pri-miR-126 in Diabetic Endothelial Cells
Source: Cells. 2022 Aug 25;11(17):2651. doi: 10.3390/cells11172651 (PMC9455028; doi:10.3390/cells11172651)
Supplement: Supplementary file 1 [file cells-11-02651-s001.zip › cells-1839647-supplementary/Supplementary Files/Supplementary figure legends.pdf]

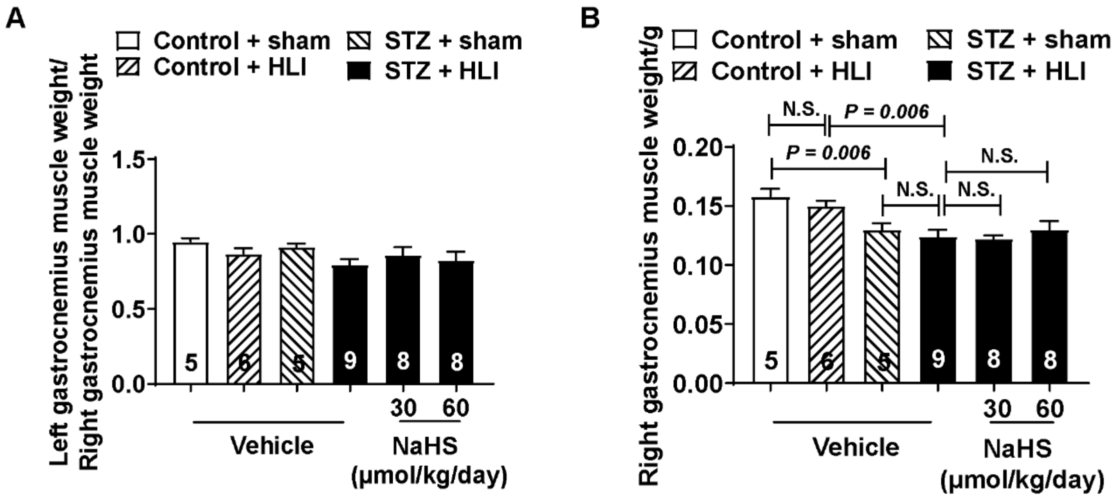

Figure S1. Muscle weight of the diabetic mice. (A) The ratio of ischemia gastrocnemius muscle weight and control gastrocnemius muscle weight with or without NaHS (30 and 60 μmol/kg/day) treatment in the diabetic mice; *n* = 5~9. (B) Control gastrocnemius muscle weight with or without NaHS (30 and 60 μmol/kg/day) treatment in the diabetic mice; *n* = 5~9.

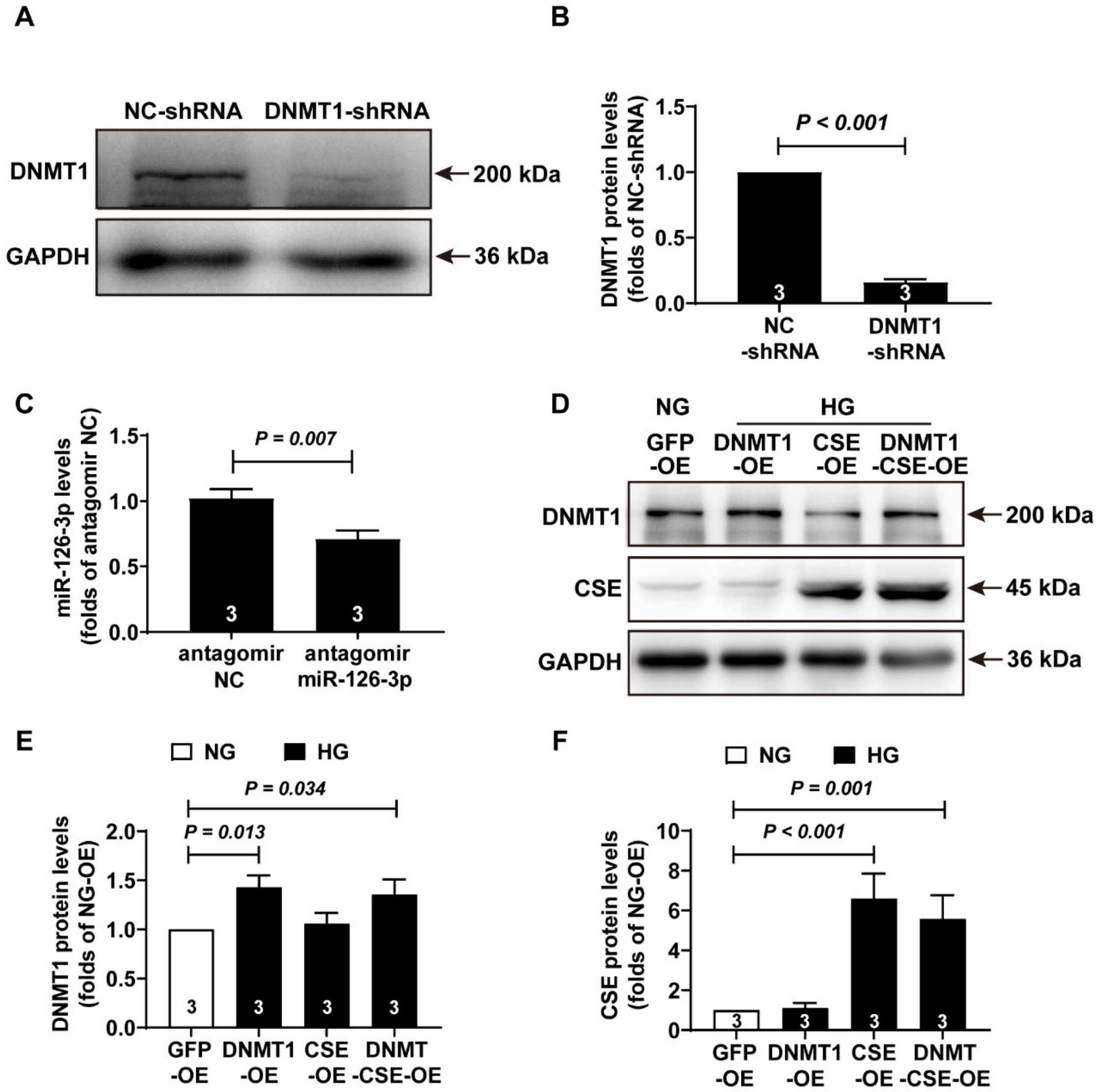

Figure S2. The efficiency of manipulating the DNMT1, miR-126-3p, and CSE expression in endothelial cells. (A,B) The inhibition efficiency of the DNMT1 protein level in HUVECs via infecting DNMT1-shRNA lentivirus; $n = 3$. (C) The inhibition efficiency of the miR-126-3p level via transfecting miR-126-3p antagomir in HUVECs; $n = 3$. (D–F) The overexpression efficiency of the DNMT1 and DNMT1 protein levels in HUVECs via infecting DNMT1-OE and CSE-OE lentivirus; $n = 3$.
