# Supplementary material for: Hydrogen Sulfide Improves Angiogenesis by Regulating the Transcription of pri-miR-126 in Diabetic Endothelial Cells
Source: Cells. 2022 Aug 25;11(17):2651. doi: 10.3390/cells11172651 (PMC9455028; doi:10.3390/cells11172651)
Supplement: Supplementary file 1 [file cells-11-02651-s001.zip › cells-1839647-supplementary/Supplementary Files/Supplementary Tables.docx]

**Supplementary information**

**Table S1. Sequences for the reverse and real-time PCR primers**

| **Primers** |  | **Sequences** |
| --- | --- | --- |
| miR-126-3p RT^*^ | Reverse | GTCGTATCCAGTGCAGGGTCCGAGGTATTC  GCACTGGATACGACCGCATTA |
| RNU6B RT^*^ | Reverse | CTCGCTTCGGCAGCACA |
| miR-126-3p^#^ | Forward | TGAGACTCGTACCGTGAGTAA |
|  | Reverse | GTGCAGGGTCCGAGGT |
| RNU6B^#^ | Forward | AACGCTTCACGAATTTGCGT |
|  | Reverse | CTCGCTTCGGCAGCACA |
| Mus-DNMT1^#^ | Forward | AAGAATGGTGTTGTCTACCGAC |
|  | Reverse | CATCCAGGTTGCTCCCCTTG |
| Mus-GAPDH^#^ | Forward | AGGTCGGTGTGAACGGATTTG |
|  | Reverse | TGTAGACCATGTAGTTGAGGTCA |
| Has-pri-miR-126^#^ | Forward | AGAAGGCAGAAGTGCCCCGTCC |
|  | Reverse | GGTCTCAGCGGCGTTTTCGATG |
| Mus-pri-miR-126^#^ | Forward | AGCCAGTTGAGTGAAAGAGCCC |
|  | Reverse | TTCCTGGCGCTGGGCTGCTG |
| MeIP gene^#^ | Forward | GGGCTCACGGGGACCCTGTCTC |
|  | Reverse | CTGACATGCTGATGTGTGGCCGA |

^*^ The primers used for reverse transcription

^#^ The primers used for real-time PCR

**Table S2. Blood glucose with or without STZ treatment**

| Group | Blood glucose fasting 8 h before treated with STZ (mmol/L) | Blood glucose fasting 4 h after treated with STZ for 2 weeks (mmol/L) |
| --- | --- | --- |
| Control+sham Vehicle | 5.18±0.14629 | 7.92±0.63119 |
| Control+HLI Vehicle | 5.2167±0.30813 | 7.45±0.25265 |
| STZ+sham Vehicle | 5.18±0.14629 | 23.04±2.65341^**^ |
| STZ+HLI Vehicle | 5.77±0.34223 | 21.26±2.34195^##^ |
| STZ+HLI NaHS (50 μmol/kg/day) | 5.1±0.34075 | 20.7222±1.77981 |
| STZ+HLI NaHS (100 μmol/kg/day) | 4.975±0.22815 | 20.25±2.58752 |

^**^ *p < 0.01* *vs* Control+sham Vehicle ^##^ *P* < *0.01* *vs* Control+HLI Vehicle

**Table S3. Body weight with or without STZ treatment**

| Group | Body weight before treated with STZ(g) | Body weight after treated with STZ for 2 weeks(g) |
| --- | --- | --- |
| Control+sham Vehicle | 24.24±0.82741 | 25.56±0.8565 |
| Control+HLI Vehicle | 23.8833±0.44378 | 25.25±0.54513 |
| STZ+sham Vehicle | 23.86±0.46648 | 22.08±0.77032^**^ |
| STZ+HLI Vehicle | 24.07±0.20979 | 22.5±0.49193^##^ |
| STZ+HLI NaHS (50 μmol/kg/day) | 23.6±0.23979 | 22.3778±0.24595^##^ |
| STZ+HLI NaHS (100 μmol/kg/day) | 23.275±0.30516 | 22.225±0.55573^##^ |

^**^ *p < 0.01* *vs* Control+sham Vehicle ^##^ *P* < *0.01* *vs* Control+HLI Vehicle
